# Production of Biopolymeric Microparticles to Improve Cannabigerol Bioavailability

**DOI:** 10.3390/ma17174227

**Published:** 2024-08-27

**Authors:** Lucia Baldino, Sonia Sarnelli, Mariarosa Scognamiglio, Ernesto Reverchon

**Affiliations:** Department of Industrial Engineering, University of Salerno, Via Giovanni Paolo II, 132, 84084 Fisciano, Italy; lbaldino@unisa.it (L.B.); ssarnelli@unisa.it (S.S.); mrscogna@unisa.it (M.S.)

**Keywords:** Cannabigerol, polyvinylpyrrolidone, supercritical CO_2_-assisted atomization, microparticles, nanoparticles, bioavailability

## Abstract

Cannabigerol’s (CBG) therapeutic effects are limited by its poor water solubility and low dissolution rate. To improve these properties, supercritical CO_2_-assisted atomization (SAA) was applied to produce coprecipitates, i.e., CBG nanoparticles coprecipitated in polyvinylpyrrolidone (PVP) microparticles. The experiments were performed by varying the CBG/PVP mass ratio (R) and the overall concentration of solutes CBG+PVP to study the influence of these parameters on particle morphology, particle size, and size distribution. Periodic dynamic light scattering (DLS) analysis was performed at regular time intervals to measure the size of CBG nanoparticles in PVP microparticles. It showed that CBG nanoparticles down to 105 nm were successfully produced through SAA. Dissolution tests were used to verify that a reduction of CBG particle size significantly increased its dissolution rate. In the liquid medium adopted, untreated CBG powder was released in 210 min, whereas CBG nanoparticles of 105 nm were completely dissolved in only 15 min.

## 1. Introduction

It is well-known that many cannabinoids can be extracted from *Cannabis sativa* L.; scientific studies about their therapeutic activity have been performed and are in progress [1]. Among them, two non-psychotropic cannabinoids emerge: cannabidiol (CBD) and Cannabigerol (CBG). CBD is the most abundant cannabinoid [2]; studies on CBD have demonstrated that it can exhibit anti-inflammatory, antioxidant, analgesic, and anticonvulsant properties [3]. CBG is regarded as a “secondary cannabinoid”, as its concentration in the cannabis plant is generally low (1% *w*/*w*) [4]. However, CBG is the precursor of various cannabinoids; consequently, it is quickly transformed in tetrahydrocannabinol (THC), CBD, and cannabichromene (CBC) [5,6,7]. CBG also shows very interesting characteristics that justify the large interest in its potential medical application [8,9]. In addition to biological and anti-inflammatory activities, CBG also exhibits antibacterial and antifungal effects [10]. It can reduce cell proliferation in several types of cancer (e.g., breast, prostate, and skin), and it has therapeutic potential in the treatment of neurologic illnesses [10,11]. Recently, a specific cultivar was produced [12] in which the fraction of Cannabigerol is very high at about 5% *w*/*w*. This opportunity discloses the possibility of producing large quantities of CBG [13,14,15,16]. However, the poor water solubility and the high hydrophobicity of CBG hinder its utilization as a nutraceutical and therapeutic agent [17,18]. To overcome these limitations, a possible solution is the reduction of CBG’s particle size, as this can improve its dissolution rate and bioavailability by increasing the exposed surface area.

Some conventional processes have been proposed to increase cannabinoids’ bioavailability, including spray-drying, solvent evaporation, homogenization, and nanoprecipitation [19,20,21,22]. On the other hand, the literature on CBG bioavailability increase is not widely diffused. Monou et al. [17] developed nanoparticles of CBD and CBG incorporated in a polymeric carrier, Pluronic-F127 (PF127). For preparative purposes of cannabinoid nanoparticles, a fixed amount of CBD or CBG and PF127 were dissolved in ethanol. After the ethanol evaporation under reduced pressure, the viscous liquid, consisting of the cannabinoid and the polymer, was added to water. CBD and CBG particles showed nano-sized dimensions, with diameters of 197 nm and 229 nm, respectively. In vitro release of CBD and CBG revealed that CBD was released within 10 h, whereas CBG was released in 24 h.

However, a first drawback associated with conventional processes is the use of large quantities of organic solvents that need to be removed [23,24,25]. Additionally, these techniques are not suitable for organic solvents forming azeotropes with water, and they are characterized by reduced control over particle size and particle size distribution [26,27,28].

Therefore, the aim of this work was the production of CBG nanoparticles encapsulated in polyvinylpyrrolidone (PVP) microparticles through supercritical CO_2_-assisted atomization (SAA), which is an alternative process to produce coprecipitates formed by polymeric microparticles in which the active principle is uniformly dispersed (nano-in-micro) [29,30]. The use of supercritical CO_2_ overcomes the limitations associated with conventional processes [26,27,28]. Indeed, SAA is industrially scalable and suitable for producing microparticles with regular morphology, controlled particle size and size distribution, and high encapsulation efficiency of the active principle [31,32]. Previous work by our research group demonstrated that PVP-K10 is a good biopolymer to produce coprecipitates through SAA because it has a relatively low molecular weight, it is water-soluble, and it can enhance the dissolution rate of hydrophobic drugs [32,33,34]. It is also non-toxic, biocompatible, and approved by the Food and Drug Administration (FDA), and it can inhibit the growth of coprecipitated materials [33,35]. CBG nanoparticles coprecipitated in PVP microparticles were thus produced, changing the CBG/PVP mass ratio (R) and the overall concentration of solutes (C_o_), to understand the effect of these parameters on particle morphology, mean size, and size distribution. The release of CBG was tested to verify modifications in its dissolution rate.

## 2. Materials and Methods

### 2.1. Materials

**Figure 1:**
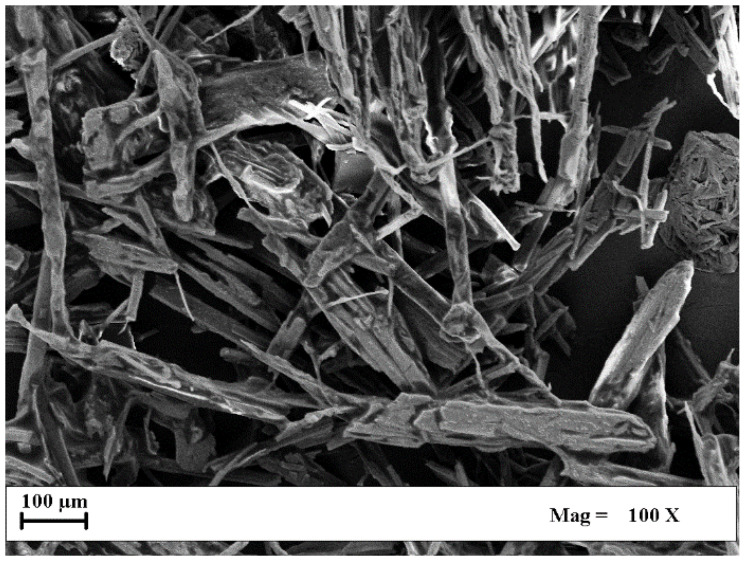
SEM image of pure CBG powder, as received.

Cannabigerol (CBG, 98% purity) was supplied by Veridia Italia Srl (Pescara, Italy); polyvinylpyrrolidone (PVP-K10, Mw = 10.000 g/mol) was purchased from Sigma Aldrich (Milan, Italy). Ethanol (EtOH, purity ≥99.9%) was purchased from Carlo Erba Reagents (Cornaredo (MI), Italy); carbon dioxide (CO_2_, >99% purity) was purchased from Morlando Group Srl (Naples, Italy); and nitrogen (N_2_, 99.9% purity) was purchased from SOL (Naples, Italy). Figure 1 shows a SEM image of CBG powder, as received, demonstrating its organization in a crystal-like structure.

### 2.2. Methods

#### 2.2.1. SAA Lab-Scale Plant

Briefly, the SAA plant consists of two high-pressure pumps (Gilson, mod. 305) delivering the liquid solution and carbon dioxide to the saturator, which is a high-pressure static mixer with an internal volume of 50 cm^3^ filled with stainless-steel 5 mm perforated saddles (Sigma Aldrich, 316 SS, mod. Z21, 053-6). The gas-expanded liquid obtained in the saturator is atomized through an injection nozzle with a thin internal wall of 80 µm in a precipitator (300 cm^3^ internal volume) that operates at atmospheric pressure. A warm nitrogen flow, stored in a tank and heated using an electric heat exchanger (Watlow, mod. CBEN 24G6), favors the evaporation of liquid droplets. A pressure gauge (Omet, mod. Cube 25, full scale 250 bar) is used to measure the pressure; thin band heaters (Watlow, mod. STB3EA10) are used to electrically heat the saturator and the precipitator. The apparatus is completed by a stainless-steel filter located at the bottom of the precipitator that allows the gaseous stream to exit and the powder to be collected. The liquid solvent is recovered using a condenser located at the bottom of the precipitator. Previous studies provide additional information about the experimental procedure and the layout of the SAA apparatus [36,37,38]. All of the experiments were performed in triplicate.

#### 2.2.2. Preparation of CBG–PVP Solution

CBG nanoparticles coprecipitated in PVP microparticles were obtained by using various mass ratios of CBG/PVP (R) equal to 1/4 *w*/*w*, 1/3 *w*/*w,* and 1/2 *w*/*w* and three different values of the total concentration of the two solutes (i.e., CBG and PVP) corresponding to 10, 20, and 30 mg/mL. For each experiment, a fixed amount of CBG and PVP was dissolved in 100 mL of ethanol, used as the liquid solvent. The operating conditions were chosen based on previous SAA experiments involving PVP as a carrier, as follows: temperature and pressure of 80 °C and 80 bar, both in the saturator and in the precipitator, and gas-to-liquid ratio (GLR) equal to 1.8 on mass basis [36,37,38].

#### 2.2.3. Characterizations

Particles’ morphology was observed using a field emission scanning electron microscope (FE-SEM, Carl Zeiss, mod. Supra 35). Before the analysis, powder samples, collected directly from the filter of the SAA apparatus, were dispersed on a carbon tab previously stuck to an aluminum stub. Then, the samples were coated with a thin gold layer using a sputter coater (mod. 108 Å, Agar Scientific, Stansted, UK) to make them conductive.

Dynamic light scattering (DLS, mod. Zetasizer Nano S) analysis was carried out to obtain information about particle size and particle size distribution. However, this analysis cannot provide information about the size of CBG nanoparticles coprecipitated in PVP microparticles. For this reason, DLS measurements were performed by operating in a periodic manner. This technique was successfully applied in a previous study [39], and it consists of observing DLS traces at different times. To perform this measurement, the sample (approximately 10 mg) was suspended in 1 mL of water and left in the DLS apparatus for the entire measurement. In this way, it was possible to follow the appearance of the CBG peak and the disappearance of the PVP peak.

The dissolution kinetics of non-processed and SAA-processed samples were measured using a UV-Vis spectrophotometer (Agilent Technologies (Santa Clara, CA, USA), mod. Cary 60 UV-Vis) using 100 mL of a solution of 50% (*v*/*v*) distilled water and 50% (*v*/*v*) ethanol as the release medium. A fixed amount of sample (i.e., 0.5 mg) was introduced in a porous sack and immersed in the liquid medium. The system was kept at room temperature and continuously stirred at 100 rpm using a magnetic stirrer. The absorbance of CBG was read at 210 nm [40]. All of the analyses were performed in triplicate.

## 3. Results and Discussion

The SAA process is based on the dissolution of supercritical CO_2_ in the solution containing the carrier (i.e., PVP) and the active principle (i.e., CBG). A gas-expanded liquid is formed in the saturator that is then atomized in the precipitator, in which a flow of warm nitrogen ensures the evaporation of the solvent from the droplets. As a result, microparticles are produced that can be collected at the bottom of the apparatus. When coprecipitation occurs, each droplet is converted into a microparticle formed by the carrier and a homogeneous dispersion of the active compound [36,37,38,39].

In this work, the CBG/PVP mass ratio and the CBG concentration in PVP were selected as the process parameters to be tested; in previous studies, it was demonstrated that they are the principal parameters controlling the final dimensions of coprecipitates [36,37,38,39]. Ethanol was chosen as the dissolving medium because CBG has a low solubility in water, as also confirmed by the literature [41], whereas its solubility in EtOH is 30 mg/mL. PVP-K10, chosen as the biopolymeric carrier, has a low viscosity that is a relevant property for the atomization process. The operating conditions selected for the experiments were 80 °C and 80 bar, as reported in the Section 2 and previously successfully used in SAA studies [36,37,38,39].

### 3.1. Effect of the CBG/PVP Mass Ratio

The first set of SAA experiments was performed at a total concentration of 10 mg/mL and by varying the CBG/PVP (R) mass ratio from 1/4 *w*/*w* to 1/2 *w*/*w*. The results, expressed in terms of the microparticle mean diameter and the standard deviation obtained through DLS analysis, are summarized in Table 1.

Generally speaking, upon increasing the amount of solubilized CBG in the solution, the mean diameter of microparticles decreased.

**Figure 2:**
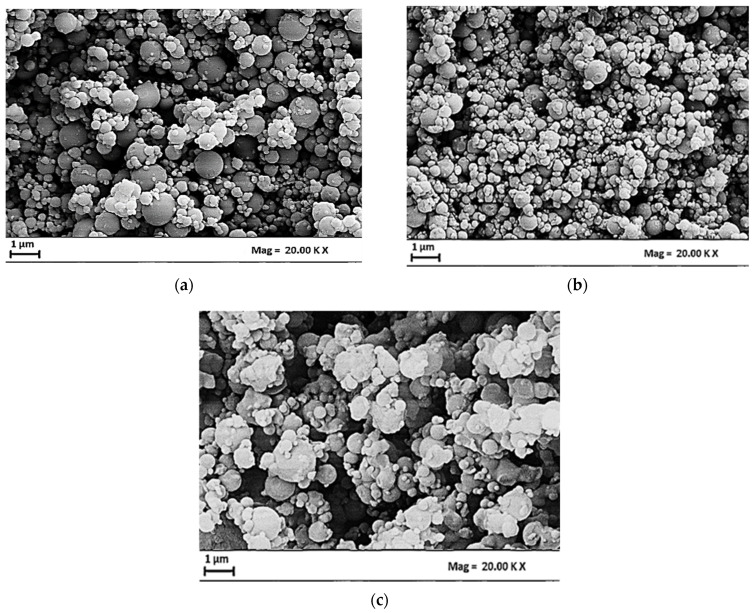
SEM images of CBG–PVP microparticles related to C_tot_ = 10 mg/mL and different values of R: (**a**) 1/4 *w*/*w*, (**b**) 1/3 *w*/*w*, (**c**) 1/2 *w*/*w*.

Examples of SEM images of CBG–PVP microparticles obtained at different R values are shown in Figure 2a–c. From a morphological point of view, an increase in the CBG/PVP mass ratio produced less-spherical microparticles. This behavior could be related to the presence of CBG, as CBD-PVP microparticles produced in another study did not exhibit this characteristic [42]. Therefore, a partial collapse of the particles, which was particularly evident when R increased, can be correlated to the increase in CBG content in the biopolymeric system.

**Figure 3:**
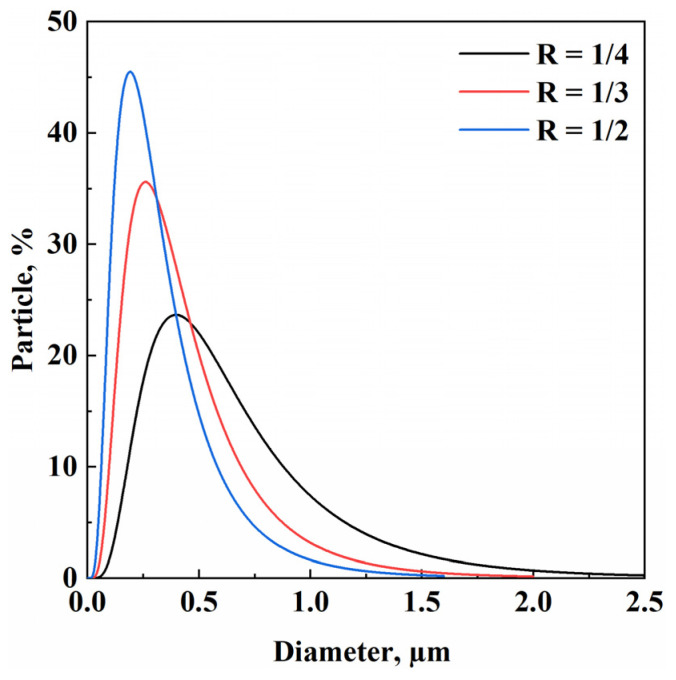
DLS results related to CBG–PVP microparticles obtained at different R values (*w*/*w*).

Figure 3 compares the size distribution of the microparticles (PSD) related to the samples S1, S2, and S3. An increase in the CBG fraction caused a reduction of the mean diameter of PVP microparticles. This result could be related to the fact that decreasing the PVP concentration led to a reduction of the main material available for growth. Consequently, the formation of new nuclei was favored over the enlargement of the existing ones.

**Figure 4:**
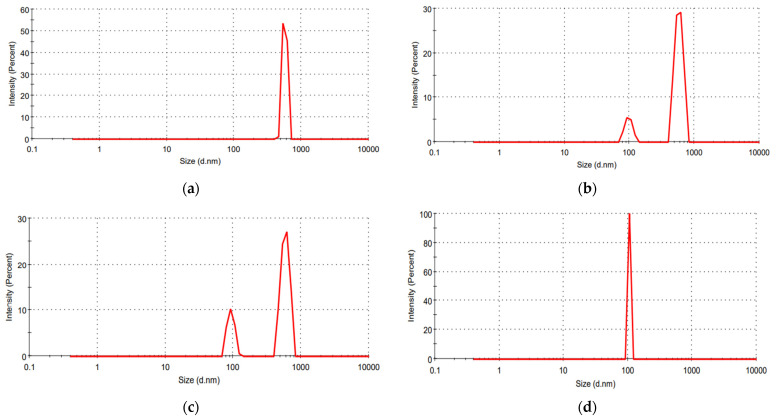
Periodic DLS for CBG–PVP microparticles obtained at R = 1/4 *w*/*w* and related to the analysis performed at different times: (**a**) t = 0 min, (**b**) t = 10 min, (**c**) t = 30 min, (**d**) t = 1 h.

Periodic DLS analysis was performed as described in the Materials and Methods for PVP microparticles obtained at R = 1/4 *w*/*w*; the related results are reported in Figure 4a–d. Briefly, periodic DLS was carried out at regular time intervals to obtain information about the size of CBG nanoparticles encapsulated in PVP microparticles. For this purpose, PVP samples were dissolved in a liquid medium in which PVP was rapidly soluble, but CBG was poorly soluble. Therefore, the DLS signal obtained at time zero referred to the mean diameter of CBG–PVP microparticles (i.e., 573 nm). After t = 10 min, the DLS trace showed two peaks because of the partial dissolution of PVP in the liquid medium; more specifically, the largest peak had an intensity of 85.6% and was related to PVP microparticles; the second one, instead, was referred to CBG nanoparticles encapsulated in PVP microparticles and showed a lower intensity equal to 14.4%. Increasing the time to t = 30 min, the intensity of the peak related to CBG nanoparticles increased up to 23.9%, whereas the intensity of PVP microparticles became 76.1%. At the end of the process, after t = 1 h, PVP was completely dissolved in the liquid medium, and the DLS trace showed only one peak related to CBG nanoparticles located at 105 nm.

### 3.2. Effect of the Total Solute Concentration

**Figure 5:**
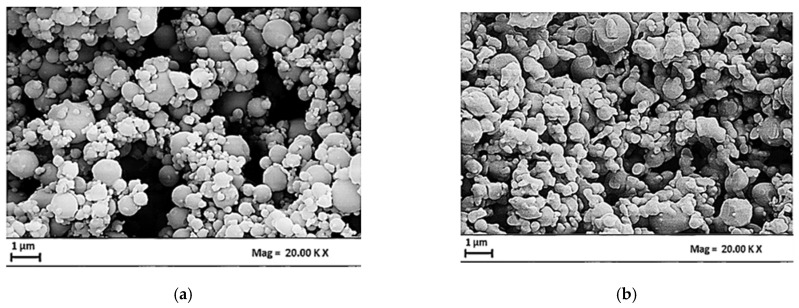
SEM images of CBG–PVP microparticles related to R= 1/4 *w*/*w* and different total concentrations: (**a**) C_o_ = 20 mg/mL, (**b**) C_o_ = 30 mg/mL.

In the second set of SAA experiments, the CBG/PVP mass ratio was set to 1/4 *w*/*w*, whereas, the overall concentration was increased to 20 mg/mL and 30 mg/mL. The morphology of the CBG–PVP microparticles was observed through SEM, as shown in Figure 5a,b.

The overall consideration is that by increasing the total concentration up to 30 mg/mL, relatively less-spherical microparticles were obtained. The measurements of the microparticles’ mean diameters and the standard deviation performed through DLS analysis are summarized in Table 2.

The effect of the total concentration on microparticle size is shown by the comparison of PSDs obtained after DLS analysis. Table 2 shows that an increase in the total solute concentration led to an increase in microparticle size; this effect was especially evident when the concentration was 30 mg/mL. This behavior was expected because by increasing the total solute concentration the viscosity of the atomized solution also increased. As a result, larger droplets were formed at the exit of the nozzle, resulting in the formation of larger microparticles [36,37,39].

### 3.3. CBG Dissolution Tests

The dissolution tests of CBG nanoparticles from PVP microparticles were carried out as described in Section 2.2.3. Parameters, such as the mean particle size, the kind of release medium (including its pH and temperature), the kind of carrier, and the active compound/carrier ratio, can affect the dissolution rate of the active compound. However, in this study, the main parameter investigated was the drug’s mean size to confirm that CBG dissolved more rapidly when its particle size decreased, thus improving its bioavailability.

The release test was performed using a mixture of water and ethanol in a 1:1 (*v*/*v*) ratio as a dissolving medium, as CBG is not particularly soluble in pure water [43]. The dissolution profiles were measured using untreated pure CBG and CBG–PVP microparticles obtained at R = 1/4 *w*/*w* and different total concentrations (i.e., 10 mg/mL and 30 mg/mL).

**Figure 6:**
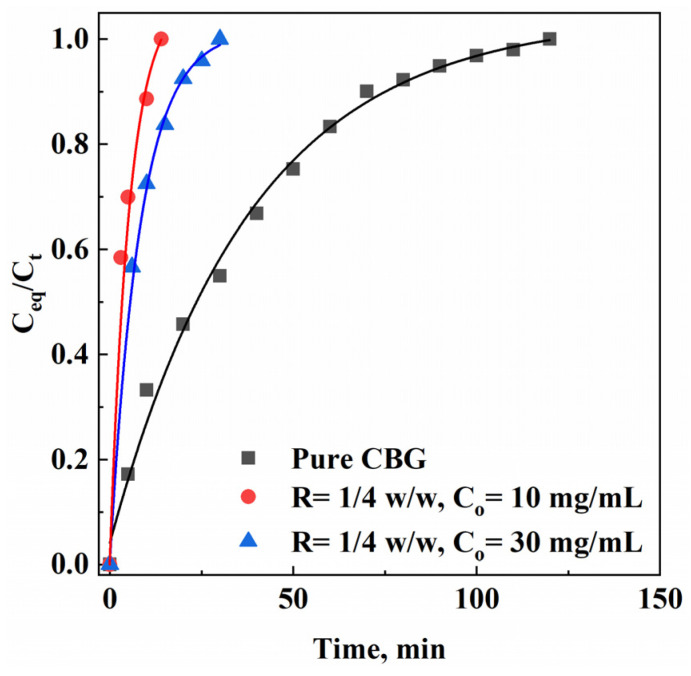
Comparison of release kinetics for untreated pure CBG powder and for CBG–PVP microparticles obtained at R = 1/4 *w*/*w* and different total concentrations (C_o_).

The results are reported in Figure 6 and expressed as C_eq_/C_t_ against time (min), where C_eq_ is the drug concentration measured at different intervals of time and C_t_ is the maximum drug concentration released in the liquid medium.

Using this dissolution medium, pure CBG powder was completely dissolved in 120 min; CBG nanoparticles of 105 nm, obtained at C_o_ = 10 mg/mL, were completely dissolved in only 15 min coming from PVP microparticles, whereas CBG nanoparticles with a diameter of 160 nm, obtained at C_o_ = 30 mg/mL, were completely released in 30 min.

These results show that the dissolution rate of CBG nanoparticles obtained at C_o_ = 10 mg/mL was very fast when compared with pure CBG and/or CBG nanoparticles obtained at C_o_ = 30 mg/mL. Smaller CBG nanoparticles dissolved faster; therefore, they can be characterized by larger bioavailability for pharmaceutical purposes. This phenomenon is primarily due to the higher surface-area-to-volume ratio of smaller particles, which provides more surface available for the dissolution process.

## 4. Conclusions

This work showed the possibility of using the SAA process to produce coprecipitates consisting of CBG nanoparticles embedded in PVP-K10 microparticles. DLS analysis was performed to obtain information on the mean size and standard deviation of the PVP microparticles produced by varying the CBG/PVP mass ratio or the overall solute concentration. To directly measure the size of CBG nanoparticles coprecipitated in PVP microparticles, a DLS analysis was also carried out in a periodic manner. The dissolution test confirmed that a reduction in particle size resulted in a shorter release time of CBG. Indeed, 105 nm CBG nanoparticles showed a reduced release time compared with the pure CBG powder.

These results are particularly relevant in the context of pharmaceutical applications, where they can lead to more effective drug delivery, lower required dosages, and reduced side effects. Consequently, CBG nanoparticles coprecipitated in PVP microparticles can be promising candidates for the development of new treatments for various diseases, such as neurodegenerative diseases, inflammatory disorders, and antibiotic-resistant infections.

## Figures and Tables

**Table 1:** DLS results referring to CBG–PVP microparticles produced at different R values.

Sample	CBG/PVP Mass Ratio	Microparticles’ Mean Diameters, nm	Standard Deviation, nm
S1	14	573	459
S2	13	379	307
S3	12	288	251

**Table 2:** DLS results referring to CBG–PVP microparticles produced at different total concentrations.

Sample	Total Concentration, mg/mL	Microparticles’ Mean Diameters, nm	Standard Deviation, nm
S1_10	10	573	459
S1_20	20	660	420
S1_30	30	771	371

## Data Availability

Data is contained within the article.
